# Preparation and Evaluation of Poly(lactic acid)/Poly(vinyl alcohol) Nanoparticles Using the Quality by Design Approach

**DOI:** 10.1021/acsomega.2c02141

**Published:** 2022-09-14

**Authors:** Meliha Ekinci, Gizem Yeğen, Buket Aksu, Derya İlem-Özdemir

**Affiliations:** † Faculty of Pharmacy, Department of Radiopharmacy, Ege University, Bornova, Izmir 35040, Turkey; ‡ School of Pharmacy, Department of Pharmaceutical Technology, Altınbas University, Bakırköy, Istanbul 34217, Turkey

## Abstract

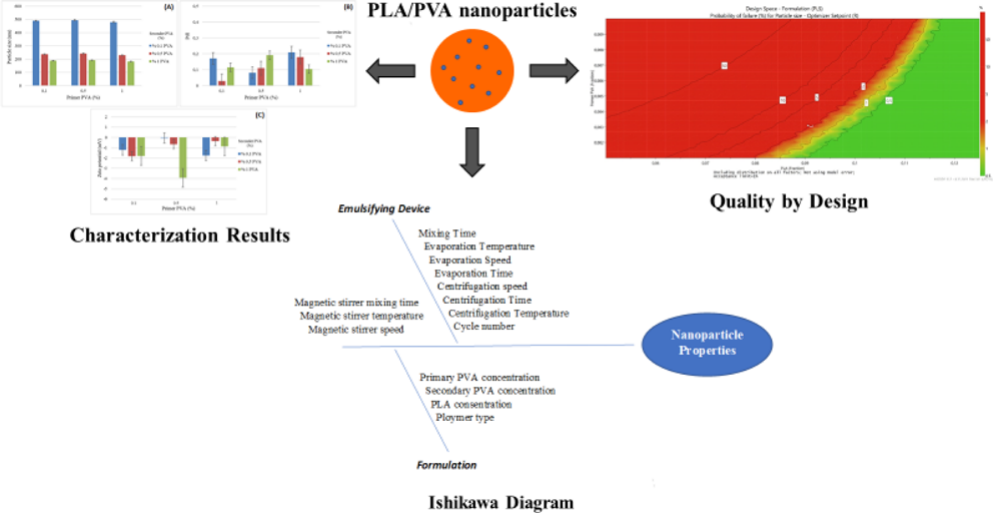

The aim of the study was to prepare and evaluate the potential use of poly(lactic acid)/poly(vinyl alcohol) (PLA/PVA) nanoparticle formulations as a drug delivery system. The nanoparticle formulations were successfully developed by the double emulsification/solvent evaporation method. The developed formulations were optimized using the quality by design approach of the ICH Q8 (Pharmaceutical Development) guideline. In the studies, the effects of emulsifying devices, evaporation technique, centrifugation effect, and polymer concentrations on the physicochemical parameters of the formulations were investigated to obtain the best results. Furthermore, the prepared formulations were evaluated for clarity, particle size, distribution, zeta potential, surface and morphological features, preparation efficiency, and long-term stability. Based on the obtained results, the nanoparticle formulation containing 12.5% PLA, 1% primer, and seconder PVA has a suitable particle size (181.7 ± 2.194 nm) and distribution (0.104 ± 0.049), zeta potential (−0.88 ± 0.45 mV), and high preparation efficiency (65.38%), and nanoparticles were spherical, had a smooth surface, and were stable up to 12 months. In conclusion, this novel formulation can be used as a potential drug delivery system.

## 1 Introduction

Nanomedicines, such as polymeric nanoparticles, nanoemulsions, and liposomes, have become increasingly popular in recent years.^1^ Polymeric nanoparticles are frequently used as drug and gene delivery systems due to their ability to protect drugs and other molecules with biological activity against the environment, their high bioavailability, safety, and biodegradability.^2^ They have been proven to accumulate preferentially at tumor sites, and their use as carriers improves efficacy while reducing side effects.^3^

In nanoparticle preparation, polymers, such as poly(-caprolactone) (PCL), poly(lactic acid) (PLA), poly(vinyl alcohol) (PVA), poly(glycolic acid) (PGA), glycolic acid copolymer (PLGA), alginate, and others, can all be employed.^4,5^ Due to the advantages of nanoparticle systems, intensive studies are carried out on the use of PLA/PVA nanoparticles as a drug delivery system.^6^ Herein, PLA/PVA nanoparticle formulations were prepared as a drug delivery system with an optimum amount and appropriate methods by selecting PLA, a renewable, biocompatible, biodegradable, and widely used polymer with good mechanical and optical properties, and PVA, a biodegradable and widely used polymer, which is water-soluble and hydrophilic, has excellent film-forming, emulsifying, and adhesive properties, and is harmless and non-toxic for living tissues.^7,8^

The International Council for Harmonization (ICH) guidelines, Q8 Pharmaceutical Development, Q9 Quality Risk Management, and Q10 Pharmaceutical Quality System, all explain the quality by design (QbD) strategy. These systems serve as the cornerstones of drug development and research. The basic goal of QbD is to develop a strategy for keeping critical formulation or process variables within an acceptable range so that product quality can be ensured while maintaining a stable and consistent manufacturing process.^9,10^

Any feature of formulation components or process parameters that has a significant impact on the desired product quality is referred to as critical. For this purpose, experimental studies should be designed in the light of risk assessment and previous scientific knowledge to determine critical variables in process and formulation.^11^

To investigate and understand the effects of critical process parameters (CPPs) and material attributes (CMAs) on product critical quality attributes (CQAs), data analyses should be performed. The design space (DS), which is a multidimensional combination and interaction of CMAs and CPPs that have been demonstrated to provide assurance of quality, should be formed to control well-understood variables.^12^

Design of experiments and mathematical models are used in the data analysis and optimization process under a QbD approach. Many statistical programs were created to help drug developers with an experimental design capability for data required during the development stage of a pharmaceutical product.^13^

The aim of this study was to prepare PLA/PVA polymeric nanoparticle formulation as a drug delivery system. For this purpose, the impact of formulation, such as primer/seconder PVA and PLA amounts, and various process parameters have on particle size, distribution, and zeta potential was enlightened, experimental results were evaluated, and then an optimum formulation was chosen via MODDE Pro (Sartorius Stedim Data Analytics) to establish DS.

## 2 Materials and Methods

### 2.1 Materials

PLA (40–100 kDa), PVA (0.1% w/v, 85% hydrolyzed), and phosphate buffer (PBS) (pH: 7.4) were purchased from Sigma-Aldrich, Germany. Dichloromethane (DCM) was purchased from Merck, Germany. The saline solution (0.9% sodium chloride solution) was obtained from Intermountain Life Sciences, LLC.

### 2.2 Defining CQAs and CMAs

**Figure 1:**
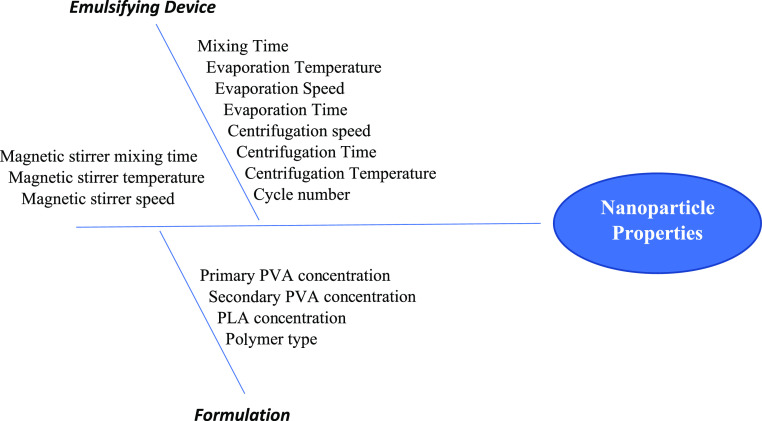
Ishikawa diagram for determining CQAs for nanoparticles. *PVA: polyvinyl alcohol, PLA: polylactic acid, and CQAs: critical quality attributes.

The parameters that could potentially affect the process performance and product were established using risk analysis.^12,14^ A fishbone diagram, as shown in Figure 1, can be used to visualize the connection between specific quality parameters. To ensure that products of the desired quality are developed, these parameters (CPP, CMA, and CQA) must be measured, analyzed, and controlled throughout the entire process.

### 2.3 Preparation of PLA/PVA Nanoparticle Formulations

PLA/PVA nanoparticles were prepared using the double emulsification/solvent evaporation method based on the method by Hernandez-Giottonini et al.^15^ The formulation was optimized by changing various parameters in the preparation of PLA/PVA nanoparticles.

In the literature reviews, it has been determined that the technique used in formulation preparation has an important effect as much as the content. For this reason, a series of controlled experiments were planned within the scope of our study.^16^ In this context, formulations with the same composition were prepared using different emulsifiers (high-speed and high-pressure homogenizer).^17,18^ Later, different volatilization techniques (rotavapor and magnetic stirrers) were used to evaporate the organic solvent in the prepared formulations.^19,20^ After examining the effect of centrifugation, which is one of the mechanical factors, the ideal preparation technique was determined. Then, using the determined ideal preparation technique, controlled experiments were carried out to determine the formulation composition. In this context, the effect of primary and secondary polymer concentrations on the physicochemical properties of formulation was investigated.^21^

#### 2.3.1 Effect of Emulsifying Devices

In order to determine the preparation method in PLA/PVA nanoparticle formulations, high-speed homogenizer and high-pressure homogenizer, which were frequently used, were used to investigate the effect of the emulsifying device.^17,18^

##### 2.3.1.1 Use of a High-Speed Homogenizer Device

For the preparation of PLA/PVA nanoparticles; first, aqueous solution of 0.6 mL of PVA (0.1% w/v, 85% hydrolyzed) was mixed with 0.6 mL of physiological saline. This mixture was added to a solution of 50 mg PLA (40–100 kDa) in 4 mL of DCM and mixed in a high-speed homogenizer (UltraTurrax T25, Ika, Germany) for different times (3, 5, and 10 min) to prepare a water/organic solvent (w/o) emulsion system. This system was then dispersed in 10 mL of PVA (1% w/v) solution. The organic solvent in the w/o/w emulsion system was obtained by mixing in the high-speed homogenizer under the same conditions (3, 5, and 10 min) was removed by evaporation under vacuum at 60 rpm at 25 °C for 1.5 h using rotavapor. Particles were recovered by centrifugation at 20,000 rpm for 20 min at 20 °C to remove excess PVA and redispersed in 2 mL of PBS (pH: 7.4).

##### 2.3.1.2 Combined Use of High-speed Homogenizer and High-pressure Homogenizer Devices

PLA/PVA nanoparticles were prepared up to the w/o/w emulsion system as described in the “Use of a High-Speed Homogenizer Device” section. Then, the w/o/w emulsion system was passed through 1 and/or 2 cycles of a high-pressure homogenizer device. Then, the organic solvent in the system was removed by evaporation using a rotavapor at 60 rpm at 25 °C for 1.5 h under vacuum. Particles were recovered by centrifugation at 20,000 rpm for 20 min at 20 °C to remove excess PVA and redispersed with 2 mL of PBS (pH: 7.4).

#### 2.3.2 Effect of Evaporation Technique

Rotavapor and magnetic stirrers were both used as evaporation techniques to investigate their impact on the preparation of PLA/PVA nanoparticle formulations.^19,20^

##### 2.3.2.1 Use of Rotavapor Device

PLA/PVA nanoparticles were prepared up to the w/o/w emulsion system as described in the “Combined Use of High-speed Homogenizer and High-Pressure Homogenizer Devices” section. Then, the organic solvent in the w/o/w emulsion system obtained by mixing in the high-speed homogenizer under the same conditions was removed by evaporation under vacuum at 60 rpm at 25 °C for 1.5 h using a rotavapor. Particles were recovered by centrifugation at 20,000 rpm for 20 min at 20 °C to remove excess PVA and redispersed in 2 mL of PBS (pH: 7.4).

##### 2.3.2.2 Use of Magnetic Stirrer Device

PLA/PVA nanoparticles were prepared up to a w/o/w emulsion system, as described in the “Combined Use of High-speed Homogenizer and High-Pressure Homogenizer Devices” section. Then, the organic solvent in the w/o/w emulsion system obtained by mixing in the high-speed homogenizer under the same conditions was removed by evaporation using a magnetic stirrer at 100 rpm at 25 °C for 1.5 h. Particles were recovered by centrifugation at 20,000 rpm for 20 min at 20 °C to remove excess PVA and redispersed in 2 mL of PBS (pH: 7.4).

#### 2.3.3 Centrifugal Effect

The effect of high-speed centrifuge on the particle size in the preparation of PLA/PVA nanoparticle formulations was investigated. For this purpose, aqueous solution of 0.6 mL of PVA (0.1% w/v, 85% hydrolyzed) was mixed with 0.6 mL of physiological saline. This mixture was added to a solution of 50 mg PLA (40–100 kDa) in 4 mL of DCM and mixed in a high-speed homogenizer for 5 min to prepare a w/o emulsion system. This system was then dispersed in 10 mL of PVA (1% w/v) solution. The organic solvent in the w/o/w emulsion system obtained by mixing in the high-speed homogenizer for 5 min was removed by evaporation using a magnetic stirrer for 1.5 h at 25 °C at 100 rpm. Then, while a group of particles was redispersed in 2 mL of PBS (pH: 7.4), recovered by centrifugation at 20,000 rpm for 20 min at 20 °C to remove excess PVA, the other group of particles was not centrifuged.

#### 2.3.4 Effect of Polymer Concentration

Because PLA and PVA concentrations are an important parameter affecting the particle size of the formulations,^21^ the physicochemical parameters (particle size, size distribution, and zeta potential) of the formulations prepared using polymers at different concentrations were examined based on the method of Hernandez-Giottonini et al.^15^

The decided formulations by mixed full factorial design were numbered by the percentage of PLA for ease of tracking. The contents of the formulations are given in Table 1.

**Table 1:** Content of Formulations

formulation	primer PVA (%)	PLA (%)	seconder PVA (%)
A1	0.1	5	0.1
A2	0.1	5	0.5
A3	0.1	5	1
A4	0.5	5	0.1
A5	0.5	5	0.5
A6	0.5	5	1
A7	1	5	0.1
A8	1	5	0.5
A9	1	5	1
B1	0.1	7.5	0.1
B2	0.1	7.5	0.5
B3	0.1	7.5	1
B4	0.5	7.5	0.1
B5	0.5	7.5	0.5
B6	0.5	7.5	1
B7	1	7.5	0.1
B8	1	7.5	0.5
B9	1	7.5	1
C1	0.1	10	0.1
C2	0.1	10	0.5
C3	0.1	10	1
C4	0.5	10	0.1
C5	0.5	10	0.5
C6	0.5	10	1
C7	1	10	0.1
C8	1	10	0.5
C9	1	10	1
D1	0.1	12.5	0.1
D2	0.1	12.5	0.5
D3	0.1	12.5	1
D4	0.5	12.5	0.1
D5	0.5	12.5	0.5
D6	0.5	12.5	1
D7	1	12.5	0.1
D8	1	12.5	0.5
D9	1	12.5	1

### 2.4 Characterization Studies of PLA/PVA Nanoparticles

#### 2.4.1 Particle Size, Distribution, and Zeta Potential Value

The prepared formulations were evaluated in terms of aggregate formation, particle size, and distribution using a Malvern Zeta Sizer in the particle size range of 3–1000 nm, at room temperature, with an angle of 173°. Samples were diluted 1:400 with filtered and bidistilled water (pH = 7) before evaluation.

The zeta potential of the formulations was evaluated using a Malvern Zeta Sizer at a field strength of 40 V cm^–1^ using a DTS 1060C zeta cuvette at 25 °C, dielectric constant of 78.5, and conductivity of 5 mS cm^–1^. Samples were diluted 1:400 with bidistilled water (pH = 7) before measuring. The mean zeta potential was determined.

#### 2.4.2 Surface and Morphological Feature Analysis Studies

##### 2.4.2.1 Scanning Electron Microscopy

The size and surface properties of PLA/PVA nanoparticles were examined under scanning electron microscopy (SEM). For this purpose, the nanoparticles were mounted onto an aluminum grid, sputter-coated with gold palladium (Au/Pd) using a vacuum evaporator, scanning of the coated nanoparticles was carried out at ×12,000 magnification and 4 kV incremental voltage conditions by a SEM device (Philips XL-30S FEG).

##### 2.4.2.2 Atomic Force Microscopy

The morphology and dimensions of PLA/PVA nanoparticles were analyzed by atomic force microscopy (AFM) to acquire topographic and phase images to investigate the diameter, height, and phase composition of the particles. For this purpose, AFM topography of PLA/PVA nanoparticles surface was taken using a Bruker Dimension Edge with ScanAsyst operating in the peak force tapping mode. The scanning tip was a silicon tip on nitride lever, 115 μm in length with a force constant of 0.4 N/m. A 10 μL of sample drop was spotted on freshly cleaved lamel. The sample solution was left on the substrates for about 1 min, blown off with air, and immediately observed by AFM.

#### 2.4.3 Preparation Yield of PLA/PVA Nanoparticles

The preparation yield of the nanoparticles was calculated gravimetrically as a percentage using eq 1.^22,23^
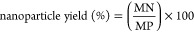
1MN: mass of produced nanoparticles and MP: mass of initial polymer materials

### 2.5 Long-Term Stability Studies of Nanoparticles

Studies to examine the stability of the developed formulations were carried out in accordance with the stability guide, at 5 ± 3 °C (in the refrigerator) and 25 ± 5 °C, 60 ± 5% relative humidity, and 40 ± 5 °C, 75 ± 5% relative humidity. In the stability study, the samples were checked for their physical appearance, particle size and distribution, and zeta potentials for 12 months at initial, 1, 3, 6, and 12 months.^24^

### 2.6 Formulation Optimization and Evaluation of DS

After characterization studies, the experimental data were evaluated using Modde Pro 12.1 (Sartorius Stedim Data Analytics) statistical computer program, which allows the optimization and establishment of a DS that is the multidimensional combination and interaction of input variables (e.g., material attributes) to provide quality assurance with response surface methodology. As seen in Table 2, the amount of primer and seconder PVA and the PLA was evaluated as a mixture/formulation factor. A mixture design was generated with 36 runs; three measurements were included as responses in the experimental design: particle size, distribution, and zeta potential value.

**Table 2:** Input Factors and Their Levels used for Specification in Modde 12.1 Pro Software

input factors	abbr.	level settings
primer PVA (%)	pri	0.1–1
seconder PVA (%)	sec	0.1–1
PLA (%)	PLA	5–12.5

The validity of the experimental design was checked using a variance test, and a mathematical model for all responses was fitted using the partial least squares regression approach in the statistical module of the Modde 12.1 Pro program (ANOVA). According to the model, an optimization process was conducted, and DS were created. Verification studies for DS were carried out with the edge point of the normal operation range in DS.

### 2.7 Statistical Analysis

Statistical analysis and variance analysis (Univariate Variance Analyze) of all the obtained characterization results were done using SPSS software version 25. The statistical significance level was accepted as *p* < 0.05 for all analyses performed. Results were obtained in triplicate and presented as the mean ± SD.

## 3 Results

### 3.1 Defining CQAs and CMAs

In Table 3, determined CQAs and their limits according to the scientific literature knowledge were given. Moreover, with risk assessment emulsifying devices, evaporation technique, centrifugation effect, and polymer concentration on the physicochemical parameters of the formulations were investigated.

**Table 3:** CQA’s for Nanoparticles

nanoparticle property	specifications
particle size	<200 nm
particle size distribution	<0.25
zeta potential	–30 mV to +30 mV

### 3.2 Preparation of PLA/PVA Nanoparticle Formulations

PLA/PVA nanoparticles were prepared successfully using a double emulsification/solvent evaporation method to examine the effect of the variables in process and formulation.

#### 3.2.1 Effect of Emulsifying Devices

The particle size, distribution, and zeta potential values of the formulations prepared by mixing at different times with the use of a high-speed homogenizer and high-pressure homogenizer devices are given in Table 4.

**Table 4:** Results of Change in Characterization Parameters of Formulations Prepared by Mixing at Different Times with the Use of High-speed Homogenizer and High-pressure Homogenizer Devices (Mean ± SD)

high-speed homogenizer (time) (min)	high-pressure homogenizer (cycle)	particle size (nm ± ss)	PdI (±ss)	zeta potential (mV ± ss)
3	-	415.8 ± 3.743	0.153 ± 0.017	–2.10 ± 0.26
3	1 cycle	373.3 ± 4.002	0.160 ± 0.025	–0.42 ± 0.35
3	2 cycles	376.2 ± 2.121	0.132 ± 0.001	–2.56 ± 0.27
5	-	290.5 ± 0.351	0.107 ± 0.083	–3.25 ± 0.10
5	1 cycle	224.4 ± 3.439	0.119 ± 0.022	–7.25 ± 0.63
5	2 cycles	223.7 ± 1.852	0.122 ± 0.052	+0.01 ± 0.26
10	-	384.7 ± 2.892	0.129 ± 0.023	–1.59 ± 0.33
10	1 cycle	273.4 ± 1.273	0.127 ± 0.008	–0.08 ± 0.10
10	2 cycles	276.2 ± 4.022	0.130 ± 0.032	–6.56 ± 0.41

Use of a high-speed homogenizer device was determined that all formulations had a homogeneous particle size distribution, and the lowest particle size was obtained after 5 min of mixing time. Furthermore, the characterization results of the particles formed as a result of only the use of high-speed homogenizer device and the combined use of high-speed homogenizer and high-pressure homogenizer device were evaluated. The particle size of the formulations prepared as a result of the combined use of the devices decreased. When the number of rounds, in which the formulations were passed through the high-pressure homogenizer device, was examined, there was no significant change in the particle size (*p* > 0.05).

Considering these results, it was decided to use high-speed homogenizer and high-pressure homogenizer devices in combination in the next studies (high-speed homogenizer device: 5 and 10 min and high-pressure homogenizer device: 1 cycle).

#### 3.2.2 Optimization of Evaporation Technique

The particle size, distribution, and zeta potential values of the formulations, in which the organic solvent was removed using the rotavapor and magnetic stirrer devices, are given in Table 5.

**Table 5:** Results of Change in Characterization Parameters of Formulations Prepared at Different Times, in which the Organic Solvent was Removed Using the Rotavapor and Magnetic Stirrer Devices (Mean ± SD)

high-speed homogenizer (time) (min)	high-pressure homogenizer (cycle)	evaporation technique	particle size (nm ± ss)	PdI (±ss)	zeta potential (mV ± ss)
5	1 cycle	rotavapor	199.7 ± 4.126	0.123 ± 0.026	–9.13 ± 1.19
5	1 cycle	magnetic stirrer	197.0 ± 4.980	0.095 ± 0.052	–0.83 ± 0.02
10	1 cycle	rotavapor	264.3 ± 6.152	0.587 ± 0.011	–1.38 ± 0.15
10	1 cycle	magnetic stirrer	230.5 ± 8.520	0.491 ± 0.039	–1.38 ± 0.34

There was no significant change in the physicochemical parameters of the formulations, in which the organic solvent was removed by using both evaporation techniques (*p* > 0.05). The use of a magnetic stirrer device was appropriate for evaporation in terms of ease of use and time method. After the evaporation process using a magnetic stirrer device, 5 min mixing time was the most suitable time because the lowest particle size was obtained after 5 min mixing time and because of the increase in PdI values of the formulations after 10 min of mixing time.

Considering these results, it was decided to use a magnetic stirrer in the next studies (high-speed homogenizer device: 5 min and evaporation technique: magnetic stirrer).

#### 3.2.3 Centrifugal Effect

The effect of centrifugation, which is carried out to ensure the recovery of the particles in the formulations, on the physicochemical parameters of the formulations was investigated. The particle size, distribution, and zeta potential values of the formulations with and without the centrifugation are given in Table 6.

**Table 6:** Results of Change in Characterization Parameters of Formulations with and without the Centrifugation (Mean ± SD)

high-speed homogenizer (time) (min)	high-pressure homogenizer (cycle)	evaporation technique	centrifugation	particle size (nm ± ss)	PdI (±ss)	zeta potential (mV ± ss)
5	1 cycle	magnetic stirrer	-	197.0 ± 4.980	0.095 ± 0.052	–0.83 ± 0.02
5	1 cycle	magnetic stirrer	20,000 rpm 20 min at 20 °C	199.6 ± 5.086	0.035 ± 0.014	–0.43 ± 0.32

The centrifugation process did not cause a significant change (*p* > 0.05) in the physicochemical parameters of the formulations, so the centrifugation process was appropriate for the recovery of particles.

Considering these results, it was decided to use the centrifugation process in the next studies.

#### 3.2.4 Effect of Polymer Concentration

**Figure 2:**
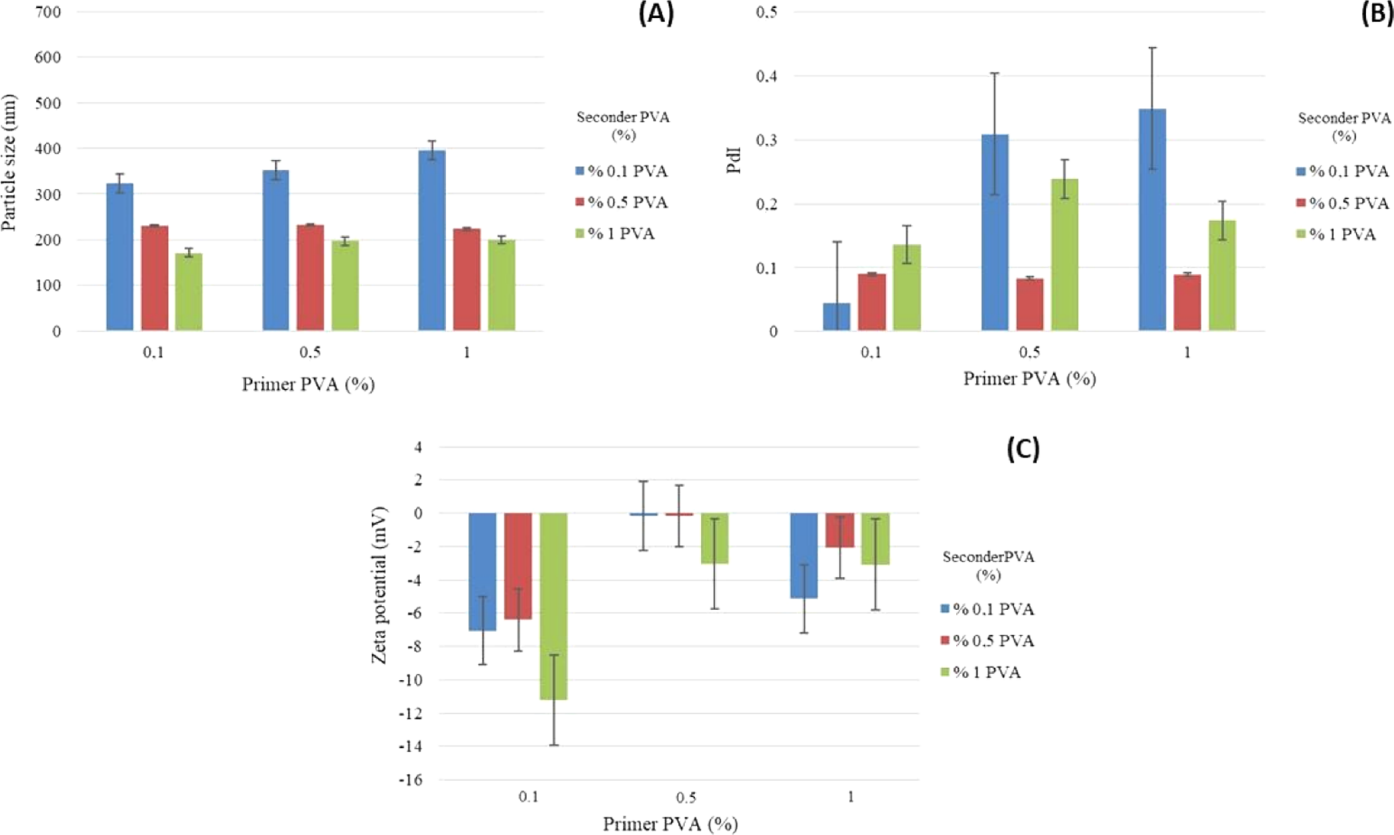
Graph of the (A) particle size, (B) PdI, and (C) ζ potential of formulations prepared using 5% PLA and different ratios of primer/seconder PVA.

**Figure 3:**
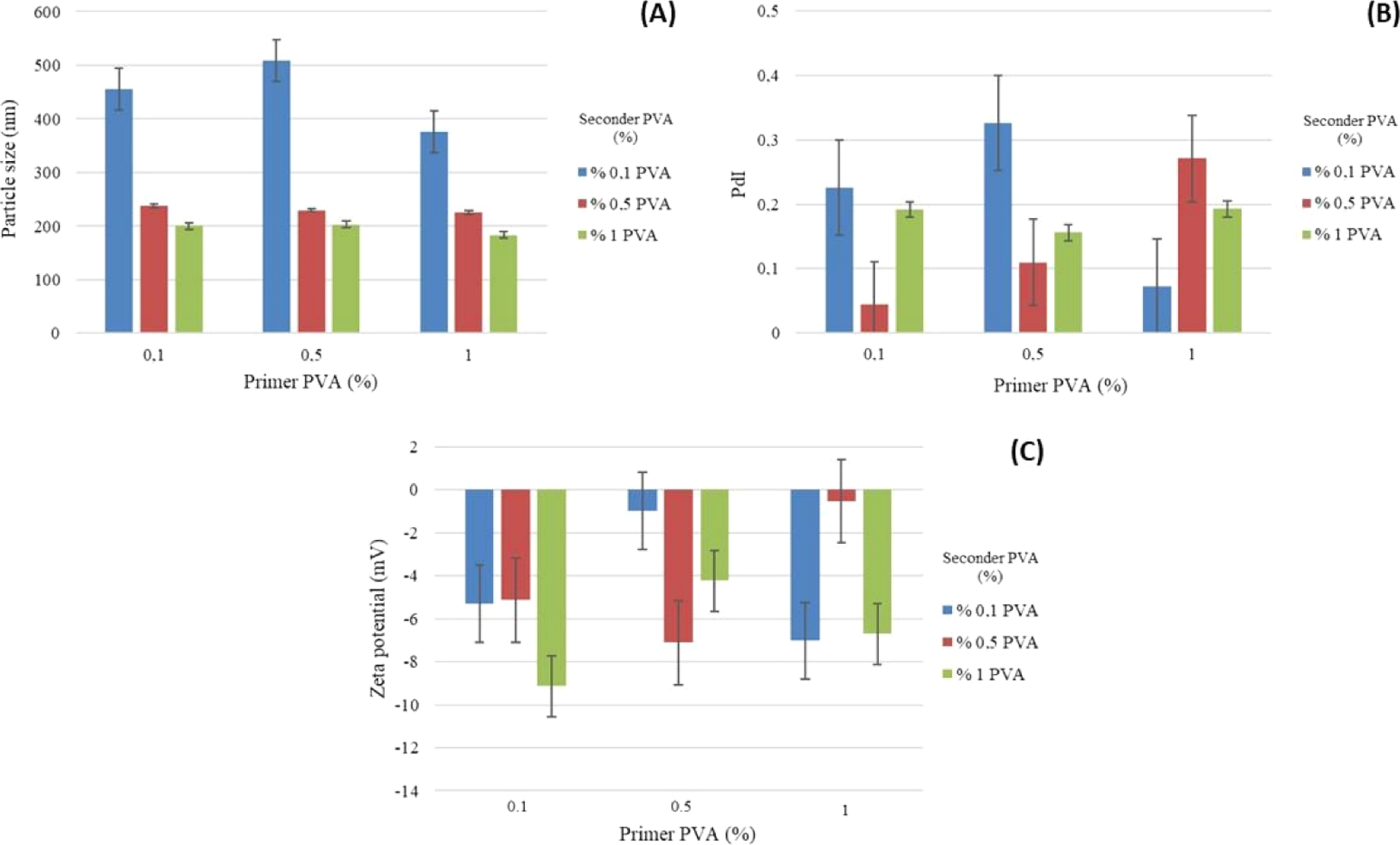
Graph of the (A) particle size, (B) PdI, and (C) ζ potential of formulations prepared using 7.5% PLA and different ratios of primer/seconder PVA.

**Figure 4:**
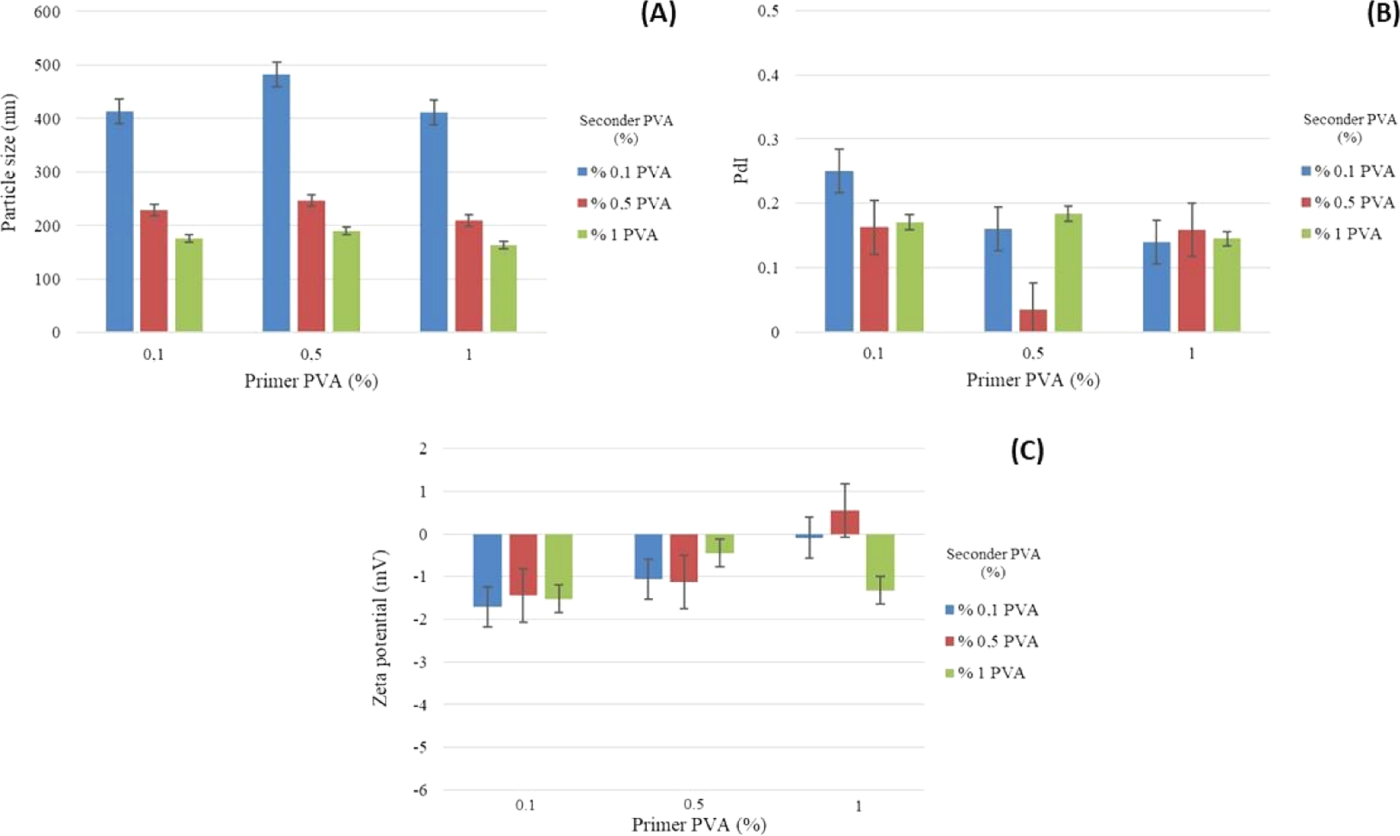
Graph of the (A) particle size, (B) PdI, and (C) ζ potential of formulations prepared using 10% PLA and different ratios of primer/seconder PVA.

**Figure 5:**
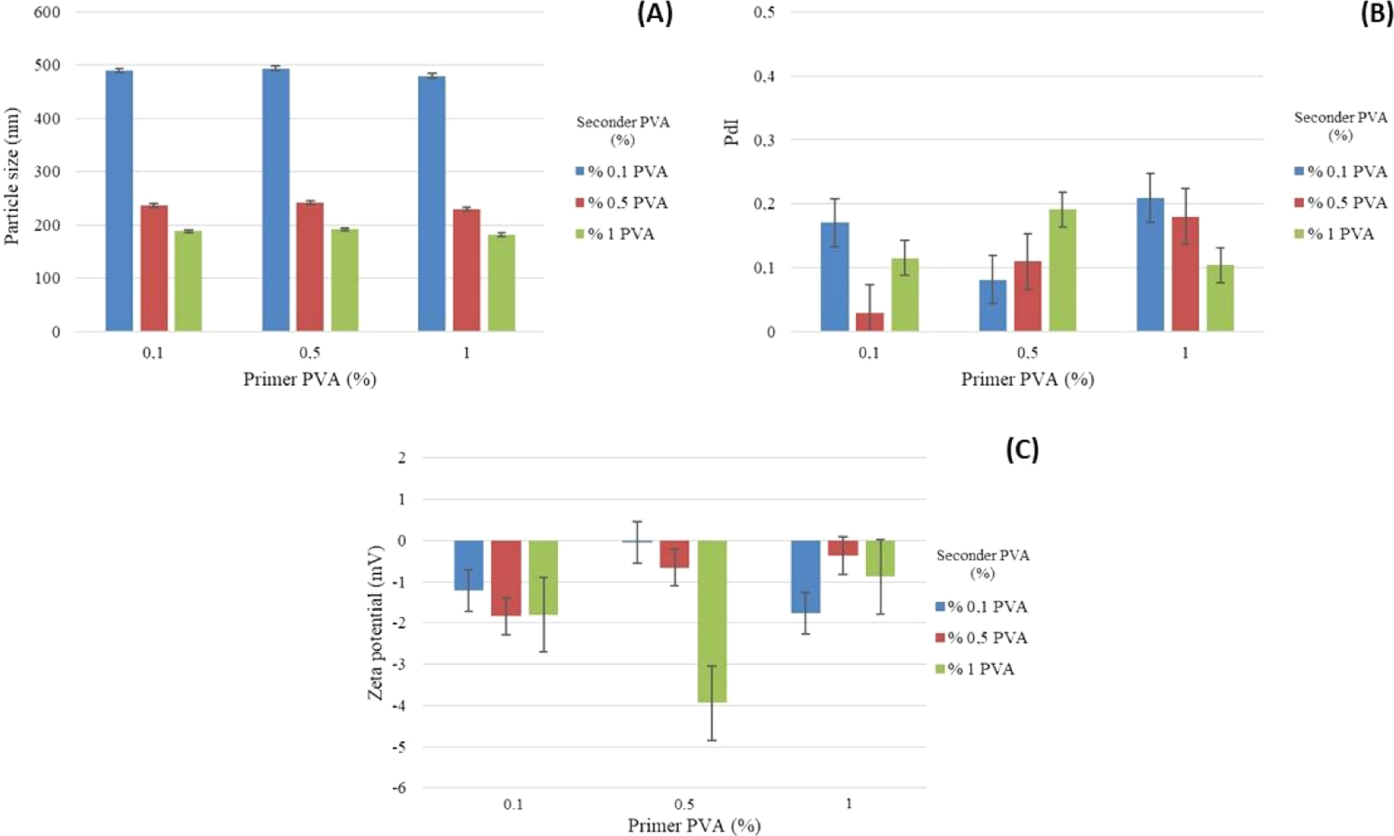
Graph of the (A) particle size, (B) PdI, and (C) ζ potential of formulations prepared using 12.5% PLA and different ratios of primer/seconder PVA.

The particle size, distribution, and zeta potential values of the formulations prepared using 5, 7.5, 10, and 12.5% PLA and different ratios of primer/seconder PVA are given in Figures 2–5.

As a result of all these studies, it was decided to perform surface and morphological analyses with formulations containing 1% seconder PVA, which were determined to have the lowest particle size.

### 3.3 Characterization Studies of PLA/PVA Nanoparticles

#### 3.3.1 Particle Size and Zeta Potential Analyses

The results of the particle size, distribution, and zeta potential analyses of PLA/PVA nanoparticle formulations are given in Figures 2–5 and Tables 4–6.

#### 3.3.2 Surface and Morphological Feature Analysis Studies

##### 3.3.2.1 SEM Analysis

**Figure 6:**
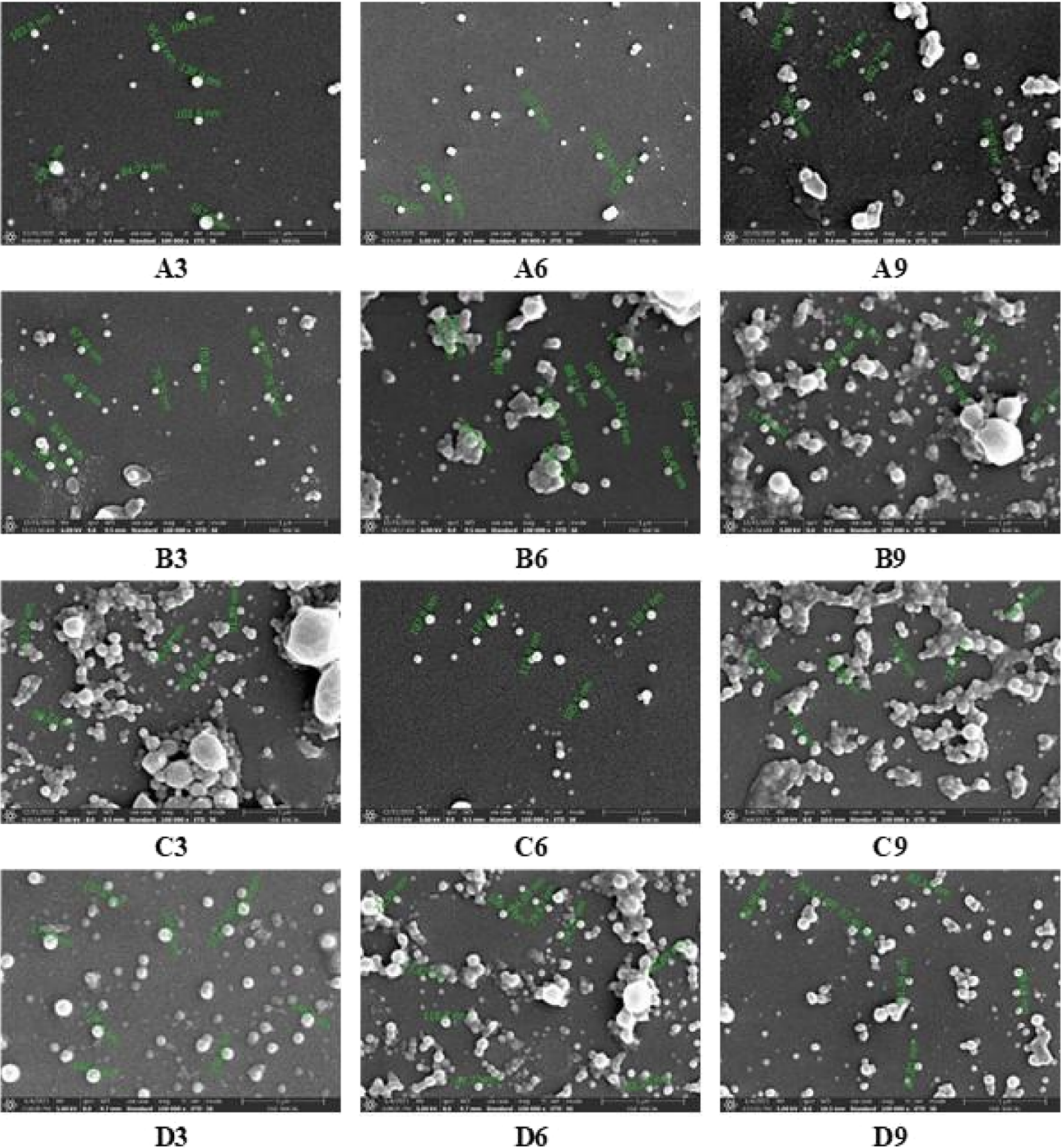
SEM images of the formulations containing 1% seconder PVA. “A”, “B”, “C”, and “D” coded formulations were prepared by using 5, 7.5, 10, and 12.5% PLA. In addition, “3”, “6”, and “9” coded formulations were prepared by using 0.1, 0.5, and 1% primer PVA, respectively.

SEM images of PLA/PVA nanoparticle formulations with ideal properties (particle size, distribution, zeta potential, and redispersibility) are given in Figure 6. The size of the particles was found to be consistent with the Malvern Zeta Sizer analysis results.

Considering these results, it was decided to use D9 formulation, which was found to have ideal properties, in the next studies.

##### 3.3.2.2 AFM Analysis

**Figure 7:**
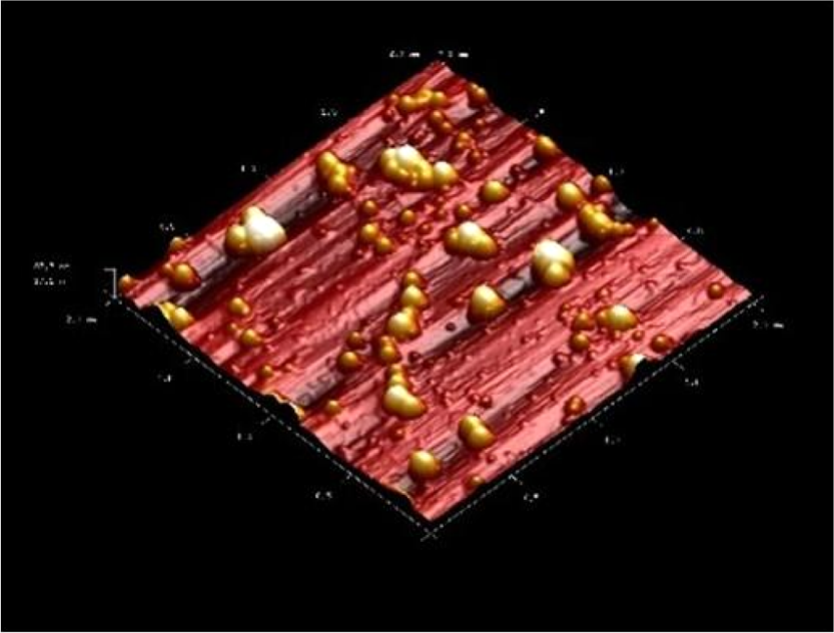
AFM image of D9 formulation.

The AFM images of the D9 formulation with ideal properties are shown in Figure 7. As seen in Figure 7, the images of the particles were found to be compatible with the SEM results. Accordingly, the particles are spherical in shape and have a smooth surface.

Advanced imaging techniques, such as SEM and AFM, can be used to determine the microscopic properties of submicron materials such as shape, size, surface morphology, crystal structure, and distribution.

#### 3.3.3 Preparation Efficiency of Nanoparticles

The preparation efficiency of D9 formulation was found to be 65.38%.

### 3.4 Long-Term Stability Studies of Nanoparticles

Stability studies of formulations stored at 5 ± 3 °C (in the refrigerator) and 25 ± 5 °C and 60 ± 5% relative humidity and 40 ± 5 °C and 75 ± 5% relative humidity for 12 months at the beginning, at 1, 3, 6, and 12 months, and the results are given in Tables 7–9.

**Table 7:** Initial, 1st, 3rd, 6th, and 12th Month Particle Size (nm ± ss), PdI, and Zeta Potential (mV ± ss) Results of PLA/PVA Nanoparticles Placed in a 5 ± 3 °C Stability Cabinet (Mean ± SD)

for.	*T*_initial_	*T*_1month_	*T*_3month_	*T*_6month_	*T*_12month_
A3	171.6 ± 2.290 nm	170.0 ± 1.607 nm	171.6 ± 6.576 nm	180.7 ± 5.147 nm	190.2 ± 3.045 nm
	0.136 ± 0.028	0.110 ± 0.058	0.162 ± 0.020	0.041 ± 0.039	0.126 ± 0.016
	–11.2 ± 1.05 mV	–0.42 ± 0.12 mV	–0.16 ± 0.15 mV	–10.6 ± 4.60 mV	–5.12 ± 0.97 mV
A6	196.9 ± 1.202 nm	211.9 ± 0.000 nm	213.3 ± 3.607 nm	226.1 ± 1.485 nm	232.2 ± 2.418 nm
	0.239 ± 0.076	0.245 ± 0.074	0.174 ± 0.103	0.148 ± 0.018	0.170 ± 0.026
	–3.06 ± 0.63 mV	–1.17 ± 0.15 mV	–1.57 ± 0.17 mV	–7.93 ± 0.71 mV	–4.38 ± 0.59 mV
A9	199.9 ± 0.353 nm	201.3 ± 5.020 nm	235.7 ± 3.404 nm	221.2 ± 1.273 nm	219.4 ± 2.109 nm
	0.174 ± 0.076	0.176 ± 0.054	0.199 ± 0.043	0.101 ± 0.094	0.156 ± 0.032
	–3.08 ± 0.46 mV	–1.37 ± 0.21 mV	–1.15 ± 0.09 mV	–6.24 ± 1.23 mV	–2.65 ± 0.75 mV
B3	199.9 ± 0.116 nm	207.1 ± 0.000 nm	219.9 ± 3.748 nm	222.1 ± 1.344 nm	222.3 ± 1.478 nm
	0.192 ± 0.038	0.182 ± 0.037	0.151 ± 0.024	0.054 ± 0.057	0.102 ± 0.041
	–9.14 ± 1.21 mV	–1.88 ± 1.15 mV	–1.69 ± 0.59 mV	–8.62 ± 1.01 mV	–5.36 ± 0.97 mV
B6	202.7 ± 3.118 nm	189.4 ± 0.778 nm	192.2 ± 2.828 nm	210.5 ± 3.260 nm	216.7 ± 3.216 nm
	0.156 ± 0.059	0.160 ± 0.066	0.073 ± 0.090	0.028 ± 0.017	0.105 ± 0.032
	–4.24 ± 0.84 mV	–1.18 ± 0.12 mV	–0.43 ± 0.16 mV	–9.03 ± 1.51 mV	–3.15 ± 0.64 mV
B9	183.6 ± 5.467 nm	176.6 ± 2.800 nm	180.8 ± 4.423 nm	197.3 ± 3.748 nm	200.6 ± 3.458 nm
	0.193 ± 0.049	0.133 ± 0.089	0.123 ± 0.074	0.051 ± 0.062	0.096 ± 0.034
	–6.71 ± 1.71 mV	–12.1 ± 2.76 mV	–2.75 ± 0.64 mV	–8.86 ± 0.71 mV	–5.36 ± 0.75 mV
C3	176.4 ± 4.172 nm	169.9 ± 0.495 nm	169.5 ± 0.473 nm	186.9 ± 2.051 nm	190.5 ± 1.432 nm
	0.171 ± 0.022	0.056 ± 0.070	0.143 ± 0.071	0.102 ± 0.047	0.112 ± 0.056
	–1.52 ± 0.40 mV	–0.25 ± 0.19 mV	–4.16 ± 0.11 mV	–7.13 ± 0.93 mV	–3.25 ± 0.41 mV
C6	189.6 ± 2.859 nm	185.9 ± 0.071 nm	187.3 ± 2.730 nm	205.1 ± 1.131 nm	198.4 ± 2.165 nm
	0.184 ± 0.012	0.126 ± 0.034	0.160 ± 0.067	0.049 ± 0.032	0.096 ± 0.018
	–0.45 ± 0.63 mV	–1.23 ± 0.10 mV	–1.23 ± 1.07 mV	–7.04 ± 1.23 mV	–3.05 ± 0.95 mV
C9	163.9 ± 2.051 nm	168.2 ± 2.121 nm	176.2 ± 1.556 nm	189.7 ± 1.697 nm	197.1 ± 1.238 nm
	0.145 ± 0.002	0.077 ± 0.028	0.175 ± 0.025	0.119 ± 0.050	0.124 ± 0.031
	–1.32 ± 0.09 mV	–1.41 ± 0.28 mV	–1.83 ± 0.11 mV	–5.11 ± 0.69 mV	–3.28 ± 0.49 mV
D3	188.6 ± 6.576 nm	192.8 ± 4.950 nm	190.1 ± 1.556 nm	198.1 ± 0.566 nm	205.4 ± 1.637 nm
	0.115 ± 0.076	0.094 ± 0.016	0.112 ± 0.052	0.243 ± 0.013	0.165 ± 0.038
	–1.88 ± 0.18 mV	+0.46 ± 0.27 mV	+0.16 ± 0.18 mV	–4.51 ± 0.34 mV	+0.87 ± 0.24 mV
D6	191.8 ± 3.111 nm	183.7 ± 3.020 nm	187.6 ± 1.273 nm	198.7 ± 1.401 nm	206.4 ± 2.665 nm
	0.191 ± 0.011	0.081 ± 0.024	0.100 ± 0.005	0.065 ± 0.047	0.054 ± 0.017
	–3.94 ± 0.28 mV	–2.19 ± 0.21 mV	–0.75 ± 0.30 mV	–6.85 ± 1.20 mV	–5.47 ± 1.39 mV
D9	181.7 ± 2.194 nm	180.0 ± 4.026 nm	197.3 ± 1.992 nm	194.5 ± 2.639 nm	195.2 ± 1.435 nm
	0.104 ± 0.049	0.158 ± 0.005	0.210 ± 0.027	0.031 ± 0.030	0.089 ± 0.036
	–0.88 ± 0.45 mV	–1.57 ± 0.65 mV	–0.31 ± 0.22 mV	–0.69 ± 0.05 mV	–1.54 ± 0.18 mV

**Table 8:** Initial, 1st, 3rd, 6th, and 12th Month Particle Size (nm ± ss), PdI, and Zeta Potential (mV ± ss) Results PLA/PVA Nanoparticles Placed in a 25 ± 5 °C Stability Cabinet (Mean ± SD)

for.	*T*_initial_	T_1month_	*T*_3month_	*T*_6month_	*T*_12month_
A3	171.6 ± 2.290 nm	170.8 ± 2.230 nm	181.9 ± 1.838 nm	186.7 ± 1.250 nm	185.6 ± 1.247 nm
	0.136 ± 0.028	0.122 ± 0.050	0.163 ± 0.011	0.163 ± 0.068	0.159 ± 0.062
	–11.2 ± 1.05 mV	+0.56 ± 0.11 mV	+0.28 ± 0.16 mV	–0.56 ± 0.29 mV	+0.68 ± 0.14 mV
A6	196.9 ± 1.202 nm	215.4 ± 0.636 nm	234.6 ± 7.508 nm	257.9 ± 10.96 nm	232.7 ± 3.245 nm
	0.239 ± 0.076	0.182 ± 0.079	0.219 ± 0.005	0.088 ± 0.035	0.106 ± 0.032
	–3.06 ± 0.63 mV	–0.35 ± 0.32 mV	–0.29 ± 0.08 mV	–1.01 ± 0.28 mV	–1.69 ± 0.14 mV
A9	199.9 ± 0.353 nm	203.4 ± 1.626 nm	211.0 ± 8.533 nm	251.2 ± 3.717 nm	241.7 ± 2.439 nm
	0.174 ± 0.076	0.219 ± 0.002	0.153 ± 0.009	0.057 ± 0.052	0.102 ± 0.028
	–3.08 ± 0.46 mV	–1.06 ± 0.16 mV	–5.47 ± 2.81 mV	+0.23 ± 0.32 mV	–2.56 ± 0.38 mV
B3	199.9 ± 0.116 nm	223.0 ± 0.849 nm	212.2 ± 2.551 nm	231.2 ± 4.140 nm	226.7 ± 3.278 nm
	0.192 ± 0.038	0.033 ± 0.004	0.181 ± 0.004	0.084 ± 0.085	0.101 ± 0.034
	–9.14 ± 1.21 mV	–0.28 ± 0.07 mV	–8.94 ± 0.64 mV	–0.47 ± 0.16 mV	–3.45 ± 0.12 mV
B6	202.7 ± 3.118 nm	196.9 ± 1.858 nm	195.5 ± 5.577 nm	212.3 ± 2.639 nm	203.4 ± 1.547 nm
	0.156 ± 0.059	0.143 ± 0.041	0.177 ± 0.049	0.074 ± 0.049	0.104 ± 0.058
	–4.24 ± 0.84 mV	–1.63 ± 0.32 mV	–5.77 ± 0.47 mV	–2.39 ± 0.91 mV	–3.17 ± 1.23 mV
B9	183.6 ± 5.467 nm	179.4 ± 2.928 nm	183.8 ± 0.707 nm	191.7 ± 2.223 nm	195.5 ± 1.234 nm
	0.193 ± 0.049	0.163 ± 0.011	0.105 ± 0.025	0.091 ± 0.044	0.103 ± 0.024
	–6.71 ± 1.71 mV	–0.11 ± 0.10 mV	–3.59 ± 0.70 mV	–0.13 ± 0.13 mV	–2.65 ± 0.34 mV
C3	176.4 ± 4.172 nm	166.2 ± 2.515 nm	162.1 ± 3.677 nm	189.8 ± 1.061 nm	190.3 ± 1.206 nm
	0.171 ± 0.022	0.143 ± 0.027	0.166 ± 0.053	0.079 ± 0.003	0.103 ± 0.024
	–1.52 ± 0.40 mV	–1.17 ± 0.69 mV	+0.35 ± 0.24 mV	–1.47 ± 0.48 mV	–1.13 ± 0.24 mV
C6	189.6 ± 2.859 nm	190.8 ± 2.987 nm	191.5 ± 3.253 nm	198.0 ± 5.940 nm	201.6 ± 3.210 nm
	0.184 ± 0.012	0.074 ± 0.066	0.073 ± 0.005	0.151 ± 0.007	0.098 ± 0.028
	–0.45 ± 0.63 mV	–0.05 ± 0.16 mV	–0.96 ± 0.48 mV	–0.71 ± 0.23 mV	+0.36 ± 0.45 mV
C9	163.9 ± 2.051 nm	175.1 ± 2.303 nm	179.2 ± 2.081 nm	189.1 ± 4.325 nm	185.3 ± 3.289 nm
	0.145 ± 0.002	0.051 ± 0.027	0.079 ± 0.087	0.062 ± 0.052	0.098 ± 0.036
	–1.32 ± 0.09 mV	–1.29 ± 0.40 mV	–0.32 ± 0.23 mV	–0.32 ± 0.44 mV	–0.56 ± 0.23 mV
D3	188.6 ± 6.576 nm	185.6 ± 2.333 nm	189.2 ± 1.762 nm	196.4 ± 3.398 nm	201.6 ± 4.214 nm
	0.115 ± 0.076	0.054 ± 0.053	0.155 ± 0.014	0.102 ± 0.101	0.087 ± 0.064
	–1.88 ± 0.18 mV	–0.47 ± 0.24 mV	–1.20 ± 0.46 mV	–0.62 ± 0.67 mV	–1.25 ± 0.61 mV
D6	191.8 ± 3.111 nm	185.5 ± 3.205 nm	186.9 ± 6.576 nm	199.6 ± 0.849 nm	203.4 ± 1.358 nm
	0.191 ± 0.011	0.129 ± 0.072	0.131 ± 0.069	0.125 ± 0.060	0.167 ± 0.053
	–3.94 ± 0.28 mV	+1.03 ± 0.31 mV	–2.30 ± 0.33 mV	+0.38 ± 0.19 mV	–1.54 ± 0.24 mV
D9	181.7 ± 2.194 nm	183.6 ± 4.649 nm	186.7 ± 3.816 nm	198.1 ± 2.192 nm	196.4 ± 1.543 nm
	0.104 ± 0.049	0.146 ± 0.026	0.082 ± 0.007	0.032 ± 0.042	0.067 ± 0.014
	–0.88 ± 0.45 mV	–0.65 ± 0.30 mV	–0.66 ± 0.47 mV	–0.87 ± 0.97 mV	–1.36 ± 0.47 mV

**Table 9:** Initial, 1st, 3rd, 6th, and 12th Month Particle Size (nm ± ss), PdI, and Zeta Potential (mV ± ss) Results of PLA/PVA Nanoparticles Placed in a 40 ± 5 °C Stability Cabinet (Mean ± SD)

for.	*T*_initial_	*T*_1month_	*T*_3month_	*T*_6month_	*T*_12month_
A3	171.6 ± 2.290 nm	165.6 ± 1.556 nm	181.5 ± 3.683 nm	182.3 ± 3.790 nm	195.4 ± 2.347 nm
	0.136 ± 0.028	0.030 ± 0.003	0.135 ± 0.055	0.122 ± 0.067	0.134 ± 0.031
	–11.2 ± 1.05 mV	–3.80 ± 0.02 mV	–7.58 ± 1.45 mV	–1.39 ± 0.53 mV	–5.21 ± 1.11 mV
A6	196.9 ± 1.202 nm	228.4 ± 0.424 nm	230.1 ± 6.718 nm	272.4 ± 15.18 nm	254.6 ± 7.230 nm
	0.239 ± 0.076	0.139 ± 0.031	0.198 ± 0.037	0.068 ± 0.058	0.154 ± 0.067
	–3.06 ± 0.63mV	–7.38 ± 0.52 mV	–0.31 ± 0.58 mV	+0.26 ± 0.51 mV	–1.37 ± 0.75 mV
A9	199.9 ± 0.353 nm	203.3 ± 2.899 nm	211.0 ± 5.398 nm	224.9 ± 0.781 nm	235.1 ± 2.430 nm
	0.174 ± 0.076	0.138 ± 0.006	0.121 ± 0.030	0.081 ± 0.015	0.134 ± 0.048
	–3.08 ± 0.46 mV	–7.94 ± 1.86 mV	–1.73 ± 0.46 mV	–4.19 ± 1.85 mV	–1.36 ± 0.39 mV
B3	199.9 ± 0.116 nm	207.7 ± 1.692 nm	217.3 ± 3.859 nm	223.1 ± 0.424 nm	215.3 ± 1.530 nm
	0.192 ± 0.038	0.124 ± 0.067	0.223 ± 0.040	0.087 ± 0.038	0.175 ± 0.023
	–9.14 ± 1.21 mV	–0.05 ± 0.06 mV	–3.04 ± 0.42 mV	–0.34 ± 0.18 mV	–1.58 ± 0.32 mV
B6	202.7 ± 3.118 nm	188.5 ± 1.706 nm	205.1 ± 0.778 nm	223.7 ± 6.788 nm	215.3 ± 4.637 nm
	0.156 ± 0.059	0.121 ± 0.032	0.138 ± 0.073	0.079 ± 0.052	0.109 ± 0.067
	–4.24 ± 0.84 mV	–3.94 ± 0.14 mV	–10.7 ± 1.77 mV	+0.31 ± 0.28 mV	–5.47 ± 1.38 mV
B9	183.6 ± 5.467 nm	174.8 ± 2.051 nm	188.4 ± 2.676 nm	201.4 ± 0.283 nm	194.3 ± 2.207 nm
	0.193 ± 0.049	0.091 ± 0.013	0.087 ± 0.047	0.038 ± 0.037	0.106 ± 0.038
	–6.71 ± 1.71 mV	–3.71 ± 0.58 mV	–6.07 ± 0.72 mV	+0.36 ± 0.13 mV	–3.27 ± 0.40 mV
C3	176.4 ± 4.172 nm	160.5 ± 0.971 nm	184.5 ± 5.706 nm	183.7 ± 4.243 nm	197.3 ± 3.210 nm
	0.171 ± 0.022	0.138 ± 0.055	0.229 ± 0.058	0.058 ± 0.021	0.145 ± 0.067
	–1.52 ± 0.40 mV	–6.05 ± 0.63 mV	–3.45 ± 0.19 mV	–1.38 ± 0.21 mV	–3.21 ± 0.27 mV
C6	189.6 ± 2.859 nm	187.4 ± 4.738 nm	200.2 ± 3.828 nm	203.9 ± 0.354 nm	217.3 ± 1.058 nm
	0.184 ± 0.012	0.139 ± 0.007	0.167 ± 0.033	0.095 ± 0.008	0.124 ± 0.052
	–0.45 ± 0.63 mV	+0.54 ± 0.32 mV	–5.87 ± 0.58 mV	+0.22 ± 0.05 mV	–1.03 ± 0.25 mV
C9	163.9 ± 2.051 nm	170.3 ± 1.345 nm	182.6 ± 3.099 nm	191.3 ± 1.061 nm	198.7 ± 2.046 nm
	0.145 ± 0.002	0.067 ± 0.048	0.093 ± 0.065	0.114 ± 0.037	0.098 ± 0.056
	–1.32 ± 0.09 mV	–6.72 ± 0.95 mV	–0.75 ± 0.36 mV	–2.35 ± 1.02 mV	–1.35 ± 0.72 mV
D3	188.6 ± 6.576 nm	182.1 ± 0.919 nm	178.9 ± 6.435 nm	198.3 ± 4.230 nm	200.3 ± 3.241 nm
	0.115 ± 0.076	0.128 ± 0.091	0.170 ± 0.016	0.088 ± 0.012	0.105 ± 0.034
	–1.88 ± 0.18 mV	–0.76 ± 0.31 mV	–1.00 ± 0.32 mV	–0.33 ± 0.10 mV	–1.07 ± 0.54 mV
D6	191.8 ± 3.111 nm	179.7 ± 4.525 nm	193.3 ± 3.126 nm	196.6 ± 2.488 nm	205.2 ± 2.685 nm
	0.191 ± 0.011	0.009 ± 0.008	0.160 ± 0.034	0.117 ± 0.095	0.087 ± 0.052
	–3.94 ± 0.28 mV	–3.26 ± 0.38 mV	–1.33 ± 0.24 mV	–0.31 ± 0.07 mV	–1.69 ± 0.32 mV
D9	181.7 ± 2.194 nm	183.9 ± 4.879 nm	193.4 ± 1.528 nm	196.3 ± 4.243 nm	198.3 ± 3.470 nm
	0.104 ± 0.049	0.150 ± 0.018	0.113 ± 0.045	0.071 ± 0.018	0.106 ± 0.024
	–0.88 ± 0.45 mV	–7.10 ± 0.73 mV	–0.80 ± 0.11 mV	–0.57 ± 0.22 mV	–1.34 ± 0.19 mV

### 3.5 Formulation Optimization and DS Evaluation

The validity of the experimental design was evaluated, mainly *R*^2^ and *Q*^2^ values given in Modde Pro 12.1. A model with an *R*^2^ (coefficient of determination) of 0.5 has a relatively low significance. *Q*^2^ (an estimation of precision of predictions) should be greater than 0.1 for a significant model and greater than 0.5 for a good model. The difference between *R*^2^ and *Q*^2^ should also be smaller than 0.3 for a good model. *Q*^2^ is the best and most sensitive indicator.

**Figure 8:**
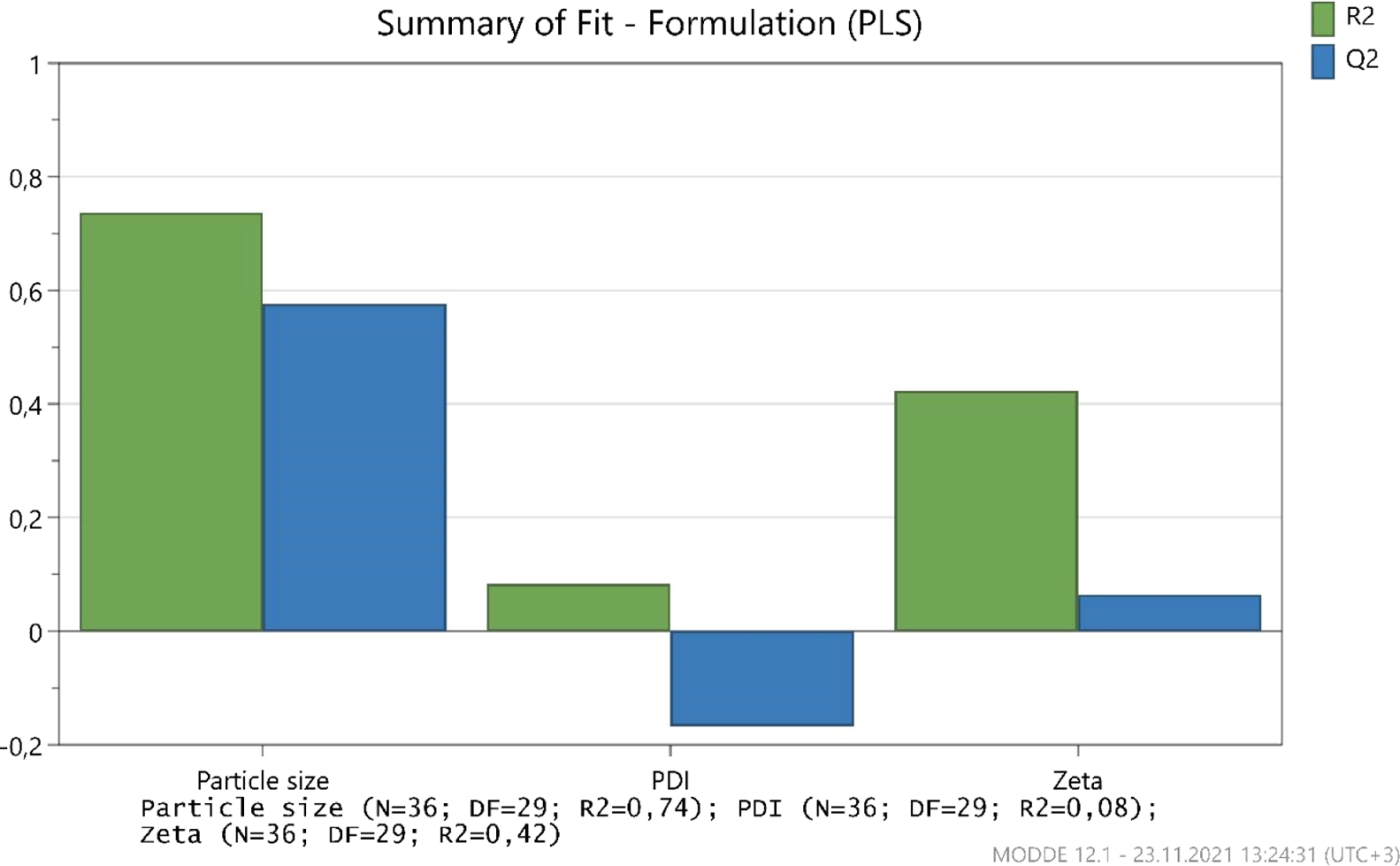
Summary of fit plot.

As seen in Figure 8, a significant model was created for only particle size (*R*^2^ is 0.74 and *Q*^2^ is 0.58). Because the main factor is the particle size in the study to search the effect of formulation parameters, optimization study was conducted according to the particle size. The revised values of the regression coefficients of the model equation are presented as a histogram for the particle size.

**Figure 9:**
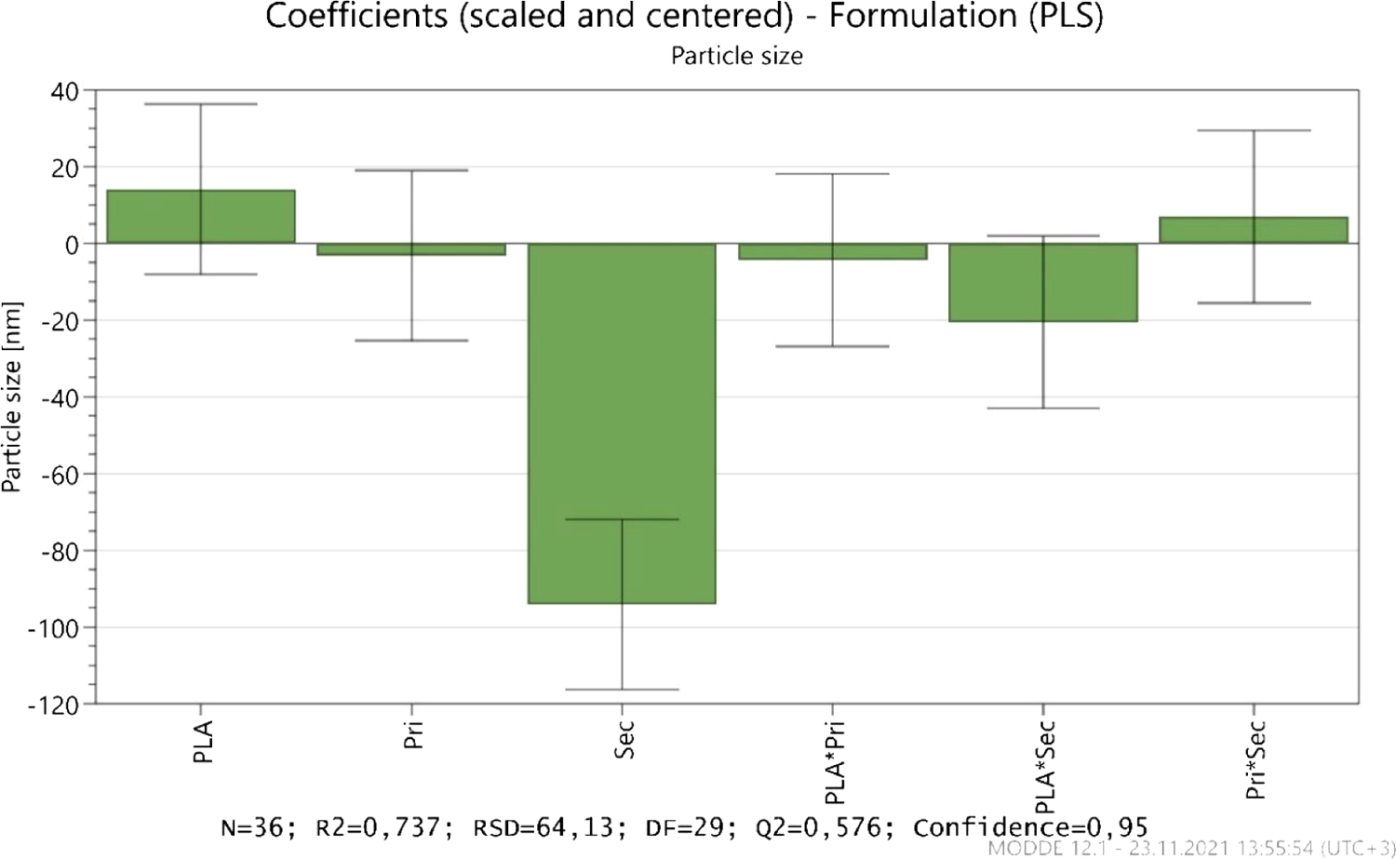
Coefficient graph demonstrates the influence of formulation variables on the particle size.

Figure 9, the coefficient plot, shows a graphical depiction of the model terms to help identify their significance and degree of variable impacts on responses. The sign of the model terms indicates whether they have a positive or negative influence on the answer. A substantial term has a large distance from *y* = 0 as well as an uncertainty level that does not cross *y* = 0.^12^

**Figure 10:**
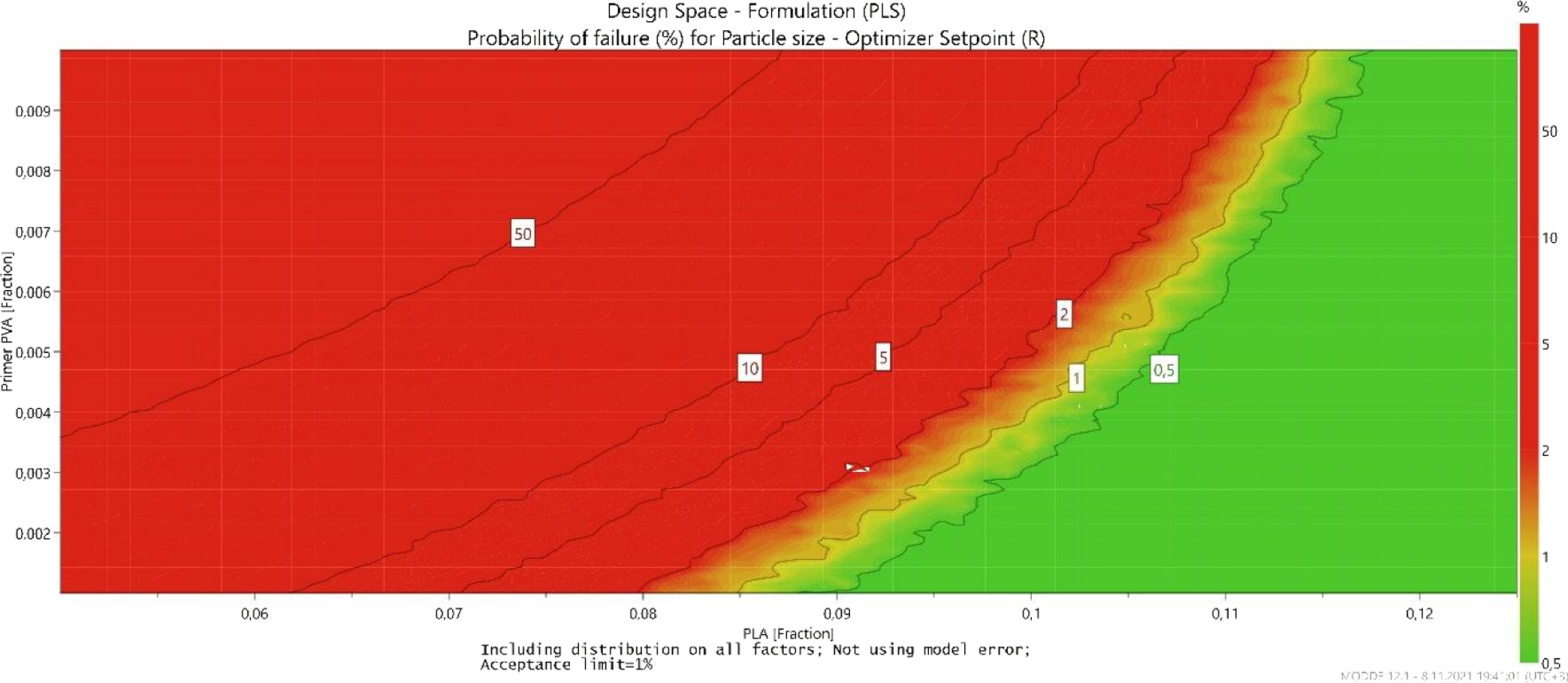
DS for the formulation variables meets the specification of the particle size.

The optimizer set points with factor settings and predicted response values, and from the initial set point chosen based on the log(*D*) (normalized distance to the target), the DS for optimal factors was generated using the robust set point function predicted by Monte Carlo simulation (resolution 64, 50,000 simulations per point, 95% confidence level), as given in Table 10.^25^Figure 10 demonstrates the DS, the green areas are part of the DS, with a less than 1% risk of failure. The regions with a higher probability of failure are depicted from yellow to red.^12^

**Table 10:** Optimizer Set Points with Factor Settings and Predicted Response Values, and the Robust Set Point of Factors

response	response objectives	optimizer initial set point	robust set point	log(*D*)	Cpk
particle size	minimize	170.97	154.96	–0.671328	0.139524
PDI	predicted	0.139783	0.140309		
zeta potential	predicted	–0.642953	–0.695698		
Factor					
primer PVA (%)		1	1		
seconder PVA (%)		1	1		
PLA (%)		12.5	12.4		

The alternative representation of the DS given in Table 11, in the case of multidimensional DS, is to describe a hypercube that defines the edges of the DS.

**Table 11:** Hypercube Edges of the DS

factors	low	high	hypercube low edge (NOP)^a^	hypercube high edge (NOP)^a^
primer PVA (%)	0.053	1.92	0.1	1
seconder PVA (%)	0.94	1.03	0.96	1
PLA (%)	11.1623	13.59	11.67	12.5
Responses				
particle size			186.3 ± 2.301 nm	181.7 ± 2.194 nm
PDI			0.212 ± 0.049	0.104 ± 0.049
zeta potential			–1.58 ± 0.21 mV	–0.88 ± 0.45 mV

a Normal operation range.

DS verification was performed by preparing the formulation edge points of normal operating range and particle size was measured, and the results are given in Table 11. All results were found to be satisfied for response requirements.

## 4 Discussion

Nanoparticles are drug delivery systems with sizes ranging from 1 to 1000 nm, which overcome biological barriers and facilitate diagnosis, treatment, follow-up of the disease and treatment responses.^26−28^ For these reasons, polymeric nanoparticle formulations consisting of PLA and PVA were prepared that can be used as drug delivery systems in this study.

In order to determine the ideal formulation, PLA/PVA nanoparticles were prepared using the double emulsification/solvent evaporation method.^15^ Some various parameters in the preparation of PLA/PVA nanoparticles have a significant effect on the physicochemical parameters of the formulation. In this context, during the formulation development studies, various parameters, such as emulsifying devices,^17,18^ evaporation technique,^19,20^ centrifugation effect, and PLA concentration,^15,21^ were changed, and ideal formulation conditions were determined.

High-speed and high-pressure homogenizer devices are frequently used in the preparation of PLA/PVA nanoparticle formulations.^17,18^ Therefore, we first carried out a series of studies to determine the ideal mixing method. The mixing time of the high-speed homogenizer device used during the formulation preparation has important effects on the physicochemical properties of the particles. All of the formulations prepared by mixing with a high-speed homogenizer at different times had a homogeneous particle size distribution, and the lowest particle size was obtained after 5 min of mixing.

The mixing time and the number of cycles that the sample is passed through the device have significant effects on the physicochemical properties of the particles, where the high-speed and high-pressure homogenizer devices used during the formulation preparation are combined. When the characterization results of the particles formed as a result of using only the high-speed homogenizer device and the combined use of both devices are evaluated comparatively; as a result of the combined use of the devices, the particle size decreased significantly (*p* < 0.05). In addition, the number of cycles of formulation passing through the high-pressure homogenizer device did not cause any significant change in the particle size (*p* > 0.05). Considering the results of the controlled experiment series, it was decided to use high-speed and high-pressure homogenizer devices in combination in formulation preparation studies (high speed homogenizer device: 5 and 10 min, high pressure homogenizer device: 1 cycle).

Because rotavapor and magnetic stirrer devices were used as evaporation techniques in the preparation of PLA/PVA nanoparticle formulations,^19,20^ the physicochemical parameters of the formulations prepared using both devices were evaluated. There was no significant change (*p* > 0.05) in the physicochemical parameters of the formulations removed by using the organic solvent rotavapor and magnetic stirrer device. Therefore, the use of a magnetic stirrer device was appropriate for evaporation in terms of ease of use and time method. In addition, because the lowest particle size was obtained after 5 min of mixing time after evaporation using the magnetic stirrer device, and there was an increase in the PdI values of the formulations after 10 min of mixing, a mixing time of 5 min was appropriate in the formulation preparation process.

The centrifugation process carried out to ensure the recovery of the particles in the formulations did not cause a significant change (*p* > 0.05) in the physicochemical parameters of the formulations, so a centrifugation process would be appropriate for the recovery of the particles.

PLA and PVA concentrations are important parameters affecting the particle size of the formulations,^15,21^ characterization studies of the formulations prepared using polymers at different concentrations were carried out. As a result of particle size and zeta potential analysis studies of PLA/PVA nanoparticle formulations, it was decided to perform surface and morphological analyses with all formulations containing 1% seconder PVA, as a decrease in the particle size was detected with an increase in seconder PVA (%), provided that the primer PVA and PLA concentrations were kept constant (A3, A6, A9, B3, B6, B9, C3, C6, C9, D3, D6, and D9).

The images obtained as a result of scanning of the PLA/PVA nanoparticle formulations coated with gold palladium on the aluminum grid are shown in Figure 7. In the images obtained, the dimensions of the particles were compatible with the Malvern Zeta Sizer results, the majority of them were spherical in shape and had a smooth surface.

The majority of the formulations had properties close to ideal in terms of stability and physicochemical properties. The QbD technique provides a lot of advantages in terms of improved scientific understanding of product and process, which allows for more regulatory flexibility and control over the manufacturing process. In terms of complex design and severe requirements for CQAs, using the QbD aspect for nanopharmaceutical products has various advantages for maximizing product performance, including particle size, zeta potential, drug loading capacity, in vitro drug release profile, surface morphology characteristics, pharmacokinetic performance, drug stability, and so forth. Therefore, QbD is increasingly becoming a valuable, widely used technique in pharmaceutical product development and manufacturing, and there are a number of studies in QbD-based nanostructured drug delivery system research.^29,30^

Therefore, in this study, the principles of QbD have been applied and experimental data were also evaluated within Modde Pro 12.1 to enlighten the multivariate relationship between critical formulation parameters and CQAs. Modde Pro 12.1 also processed optimization and created a DS throughout the data.

According to the model fitting results demonstrated with the coefficient plots,^12,31^ increasing the seconder PVA amount shows a significant effect on particle size reduction in accordance with previous knowledge of studies, while other variables and their interactions seem less effective (also with uncertainty), as seen in Figure 9.

An optimized formulation adjusted to reach the lowest particle size was suggested as 1% primer PVA and 1% seconder PVA with 12.5% PLA, which is the same as the D9 coded formulation. Within the study, DS were also observed as representing the sum of the variable combinations that lead to the desired CQA. As validation studies for DS show a robust range for ingredients that formulation meets the requirement for particle size were appointed. Considering the features, such as the program used, redispersibility after centrifugation, preparation efficiency, it was decided to continue with the D9 coded formulation.

AFM or SEM based on imaging nanoparticles is considered a reference technique for measuring the size of nanoparticles. In contrast to microscopy-based techniques, sizing techniques such as dynamic light scattering (DLS) are classified as indirect because they are the result of a computation or modeling process in size measurement.^32^ Furthermore, the AFM and SEM analyses corroborated the DLS results, confirming the quality and homogeneity of the particles.

The preparation efficiency of PLA/PVA nanoparticle formulation (D9) was calculated as 65.38%.

According to the stability studies, all PLA/PVA nanoparticle formulations were tested. It was stable and did not show a significant change in particle size, distribution, and zeta potential under all three conditions (*p* > 0.05).

The zeta potential is an extremely important concept for the stability of nanoparticle suspensions due to electrostatic repulsion between charged particles. As the zeta potential value increases in nanoparticle formulations, the stability of colloidal dispersions is ensured. Nanoparticulate formulations with a low zeta potential cannot maintain their stable state. In such cases, these nanoparticulate formulations should be stored in lyophilized form rather than liquid suspension and reconstituted just before use.^33^ The zeta potential value can influence the nanosystems to undergo phagocytosis. Nanoparticles with near-zero zeta potential undergo lower phagocytic uptake compared to nanoparticles with a positive zeta potential. As the pH of PLA/PVA nanoparticle formulations increases, carbonyl groups in PLA and hydroxyl groups in PVA settle close to the nanoparticle surface, and negative and high zeta potential values are measured.^34^ However, when the pH of PLA/PVA nanoparticles is around 7, the zeta potential value decreases and approaches zero. In our studies, the zeta potential value of almost all nanoparticle formulations was determined to be close to zero. Accordingly, it was determined that the preparation method was not effective on the zeta potential (*p* > 0.05).

## 5 Conclusions

In preformulation studies, it is known that the preparation method as well as the composition is effective on the physicochemical parameters of the formulation. In this context, we examined the effects of emulsifying devices, evaporation technique, and mechanical preparation methods, such as centrifugation on the formulation with our controlled experiments. After determining the ideal mechanical preparation method, we examined the effect of the amount of primary and secondary emulsifiers, which are the most effective factors in the double emulsification method. PLA/PVA nanoparticles were successfully developed by double emulsification/solvent evaporation method using a high-speed homogenizer device. To evaluate the effect of formulation parameters on CQAs and to optimize formulation variables by creating a DS, Modde 12.1 software was usefully applied to build a mathematical model within the framework of the QbD approach.

Adopting Qbd approach and using mathematical modeling programs in the study, to develop a nanoparticle formulation and determining the acceptable range for formulation variables to assure the required quality attributes, has improved the efficiency of the developing stage through increased product knowledge. This aspect within the QbD framework has enabled us to make decisions based on scientific and risk-based information while encouraging operational excellence.

In conclusion, the developed formulation (12.5% PLA, 1% primer and seconder PVA) has ideal properties to be used as a drug carrier system, such as particle size (181.7 ± 2.194 nm, polydispersity index (0.104 ± 0.049), zeta potential (−0.88 ± 0.45 mV), high preparation efficiency (65.38%), and long-term stability up to 12 months.
